# Local Invasion Patterns Characterized by SARIFA and Tumor Budding Differ and Have Distinct Prognostic Significance in Esophageal Adenocarcinoma and Squamous Cell Carcinoma

**DOI:** 10.3390/cancers16183144

**Published:** 2024-09-13

**Authors:** Ákos Jakab, Levente Zarándy, Ildikó Kocsmár, Tibor Várkonyi, István Kenessey, Attila Szijártó, András Kiss, Tamás Vass, Gábor Lotz, Éva Kocsmár

**Affiliations:** 1 Department of Pathology, Forensic and Insurance Medicine, Semmelweis University, Üllői Str. 93, H-1091 Budapest, Hungary; jakab.akos@stud.semmelweis.hu (Á.J.); zarandy.levente@stud.semmelweis.hu (L.Z.); varkonyi.tibor1@semmelweis.hu (T.V.); kenessey.istvan@oncol.hu (I.K.); kiss.andras@semmelweis.hu (A.K.); 2 Department of Urology, Semmelweis University, Üllői Str. 78/b, H-1082 Budapest, Hungary; kocsmar.ildiko@semmelweis.hu; 3 National Cancer Registry, National Institute of Oncology, Ráth György Str. 7-9, H-1122 Budapest, Hungary; 4 Department of Surgery, Transplantation and Gastroenterology, Semmelweis University, Üllői Str. 78, H-1082 Budapest, Hungary; szijarto.attila@semmelweis.hu (A.S.); vass.tamas@semmelweis.hu (T.V.)

**Keywords:** tumor budding, poorly differentiated clusters, SARIFA, esophageal cancer, esophageal squamous cell carcinoma, esophageal adenocarcinoma, invasion front

## Abstract

**Simple Summary:**

Tumor budding (TB), poorly differentiated clusters (PDCs), and the Stroma AReactive Invasion Front Area (SARIFA) are emerging biomarkers offering fast and cost-effective ways of assisting the clinical therapeutic decision. However, in esophageal cancers, their applicability has not been fully elucidated. In our retrospective study, we examined these invasion markers in a cohort of esophageal squamous cell cancers and adenocarcinomas, with a special focus on lymphatic spread. According to our results, different invasive patterns are prognostic in histological subtypes of esophageal cancer, namely, SARIFA in adenocarcinomas and TB in squamous cell carcinomas. However, only in squamous cell cancers were TB and PDC useful for the prediction of overall survival. In adenocarcinomas, neither of the aforementioned markers were significant regarding survival prediction, possibly due to the small cohort size.

**Abstract:**

Both esophageal squamous cell carcinoma (ESQCC) and adenocarcinoma (EAC) are known to have poor prognosis. We aimed to investigate the invasion front areas of 57 ESQCC and 43 EAC cases to find histological signs of metastatic progression. Tumor cell clusters with different cell counts, including tumor buds (TBs) and poorly differentiated clusters (PDCs), were assessed. The presence of the recently described Stroma AReactive Invasion Front Area (SARIFA) phenomenon, which defines a direct contact between tumor cells and adipocytes, was more frequently observed in EAC than in ESQCC (*p* = 0.004). In adenocarcinomas, a higher prevalence of SARIFA was observed in tumors with a higher number of small clusters (TBs and small PDCs; *p* < 0.001); furthermore, both the high number of TBs (*p* = 0.016) and the presence of SARIFA (*p* = 0.001) correlated with a higher pT stage. SARIFA positivity in EAC (*p* = 0.011) and high TB in ESQCC (*p* = 0.0006) were found to be independent prognostic factors for lymph node metastases. Moreover, in ESQCC, the higher absolute number of both TBs and PDCs was associated with shorter overall survival (*p* = 0.0269 and *p* = 0.0377, respectively). Our results suggest that the histological subtypes of esophageal cancer behave differently, namely, that different features of the invasion front are of prognostic significance.

## 1. Introduction

In Western societies, the predominant cancer entity of the esophagus is adenocarcinoma, which surpassed squamous cell carcinoma only a few decades ago [1]. This shift in trend is attributed to changes in risk factors, with a decrease in alcohol and nicotine consumption and a simultaneous increase in the incidence of obesity and gastro-esophageal reflux disease [1]. According to the data of the GLOBOCAN website for Hungary, esophageal cancer ranks 22nd in terms of incidence and 13th in mortality. The decreasing trend of squamous cell carcinomas can also be observed in Hungary, yet only 34.7% of esophageal cancers diagnosed in 2021 were adenocarcinomas, compared to 60% in Western European countries [2,3,4]. The prognosis of the two types is predicted by different prognostic markers, greatly influencing therapeutic clinical decisions. Considering their predictive value, it is important to emphasize from a cost-effectiveness and time-saving perspective that the most useful of these markers are those that can be assessed from routine hematoxylin–eosin (HE)-stained histological sections, especially if they are also obtainable from preoperative biopsies. 

One of these is tumor budding (TB), which was established as an independent prognostic factor during the International Tumor Budding Consensus Conference (ITBCC) in 2016, and since then it has been part of the guidelines of the Union for International Cancer Control Tumor Lymph Nodes Metastasis staging system (UICC TNM) for the staging of colorectal cancer [5]. TB is a solitary tumor cell or a cluster of up to four tumor cells, while its counterpart, the poorly differentiated cluster (PDC), is a cluster of more than five tumor cells (without glandular differentiation and thus, absence of lumen) [5]. However, despite the arbitrary difference, they are part of the same biological phenomenon, the epithelial–mesenchymal transition, a hallmark of tumor dissemination, when the tumor cells gain migratory potential by changing the expression of cell adhesion molecules. The loss of the epithelial phenotype is a key element of the metastatic cascade, which has a profound effect on tumor progression and thus, patient survival [6,7]. There have been many attempts to determine the applicability of TB in other tumor types as well, most commonly in upper gastrointestinal tract cancers [8]. In our previous study, we proved its usefulness in gastric adenocarcinoma, where we found it to be an independent predictor of overall survival as well as disease-free survival [9]. Tumor budding can also be assessed from biopsy specimens by examining intra-tumoral TB, which has been shown to have a significant positive correlation with budding in the invasive front (peritumoral) [10]. The budding assessed on preoperative biopsy samples may be used to predict tumor aggressiveness and thus, determine the further treatment options, including the need for neoadjuvant therapy [10]. 

Stroma AReactive Invasion Front Area (SARIFA) is a newly emerging biomarker, mostly studied on colorectal and gastric cancer [11,12,13]. It is a phenomenon where a group of at least five tumor cells are in direct contact with the adipocytes in the invasion front, without stromal or histiocytic reaction [14]. The tumor cells in SARIFAs express FABP4 (Fatty Acid Binding Protein 4) and show a higher expression of CD36, which indicate the capability of the tumor cells for taking up and breaking down fatty acids [15,16]. This suggests an underlying mechanism by which tumor cells are able to change their metabolism from glycolysis to lipid-based energy production. The CD36-expressing tumor cell population represents a metastatic-prone subgroup of tumor cells, so SARIFA positivity combined with the assessment of tumor budding gives a new perspective to evaluate the potential of metastasis development [17,18].

Frequent metastatic spread through the lymphatic vessels is particularly responsible for the poor prognosis of esophageal cancer. Therefore, we investigated the histological phenomena of tumor spread in the invasion front that are expected to determine the metastatic potential and have prognostic/predictive value. Accordingly, our aim was to investigate the prognostic significance of histologic phenomena at the invasion front, such as tumor budding (TB), poorly differentiated clusters (PDCs), and the Stroma AReactive Invasion Front Area (SARIFA) in esophageal squamous cell carcinoma (ESQCC), as well as in esophageal and esophagogastric junction adenocarcinoma (EAC).

## 2. Materials and Methods

### 2.1. Case Selection and Clinical Data

Our study was designed to retrospectively investigate the cases of patients who underwent esophageal resection for esophageal squamous cell carcinoma or esophageal/esophagogastric junction adenocarcinoma between 2008 and 2021, and whose tumor histopathological work-up was performed in the Department of Pathology, Forensic and Insurance Medicine, Semmelweis University, Budapest, Hungary. Patients with incomplete clinical data, those who died within 30 days of surgery (perioperative death), and those who went into complete remission after neoadjuvant therapy due to complete disappearance of the tumor were excluded from this study, yielding a total of 100 cases available for analysis. The detailed clinical histories and pathological data of the patients were collected from the electronic patient register of Semmelweis University and the register of the Department. Additional follow-up data were obtained from the National Cancer Registry (National Institute of Oncology, Budapest, Hungary). The most relevant clinicopathological features of the cohort are listed in Table 1. Further details of the clinical and pathological data are provided in the Supplementary Materials, Table S1.

### 2.2. Histological Work-Up and Assessment

Esophageal squamous cell carcinomas (ESQCCs) and esophageal/esophagogastric junction adenocarcinomas (EACs) were defined, and the pTNM stage was determined following the 8th edition of the UICC guidelines [19]. The evaluation of the effect of neoadjuvant therapy was based on the Mandard score [20], which describes the remnant tumor tissue in the post-neoadjuvant histological sample. Patients were classified into responder (Mandard 2, 3, and 4) and non-responder (Mandard 5) categories (cases with complete remission of the tumor after neoadjuvant therapy with a Mandard score of 1 were excluded from the study).

From each patient, multiple HE-stained sections were examined by brightfield microscopy, and the sections with clear representation of the invasion fronts were scanned and digitized (Panoramic 1000 Digital Slide Scanner, 3D Histech, Budapest, Hungary). Tumor budding (TB) and poorly differentiated cluster (PDC) counts as well as the occurrence of SARIFA were evaluated on the digital slides in CaseViewer software (version 2.4.0, 3D Histech, Budapest, Hungary), independently by two specially trained investigators (A.J. and L.Z.) blinded to clinical and outcome data. The evaluations of the tumor buds and poorly differentiated clusters were performed according to the recommendations of the International Tumor Budding Consensus Conference (ITBCC) [5]. Tumor buds were defined as a single individual tumor cell or as a solid, lumen-free group of up to four tumor cells, following the ITBCC recommendation. Accordingly, poorly differentiated clusters (PDCs) corresponding to larger infiltrating tumor cell clusters were defined as solid, lumen-free nests of five or more tumor cells [21]. The TBs and PDCs were counted in the invasive front of each tumor using the hot-spot method, in a field of 0.785 mm^2^ at a total magnification of 200×, which is considered to be the most useful approach for assessing tumor budding in colorectal cancer and is also incorporated in the ITBCC guidelines. In each case, the TB and PDC counts recorded by the two separate investigators were averaged, and the resulting TB or PDC count was used for further analysis. In case of substantially discrepant results, the given case was jointly assessed again in a second round involving further investigators experienced in tumor histopathology (É.K. and G.L.), and a consensus was reached. 

**Figure 1:**
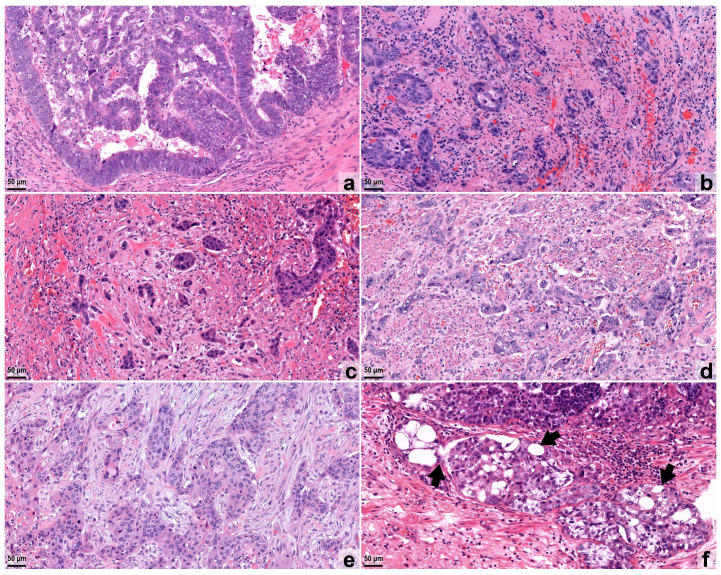
Examples of the histopathological appearance of tumor budding (TB), poorly differentiated clusters (PDCs), and Stroma AReactive Invasion Front Areas (SARIFAs) in the investigated digital slides of hematoxylin–eosin-stained sections of esophageal squamous cell carcinoma (ESQCC) and esophageal/esophagogastric junction adenocarcinoma (EAC). (**a**) “Pushing border”-type invasion pattern of an EAC with no visible tumor bud or PDC (Bd 0/PDC 0). (**b**) EAC exhibiting high tumor budding (Bd 3). (**c**) ESQCC case showing high tumor budding (Bd 3). (**d**) EAC of high PDC status (PDC 3). (**e**) Infiltration of an ESQCC with high PDC status (PDC 3). (**f**) Appearance of SARIFA (indicated by arrows) in an EAC case. Original magnification: 200×.

Based on the total number of tumor buds (TBs) in the hot-spot area, the cases were categorized into budding grades (Bd 0: 0, Bd 1: 1–4, Bd 2: 5–9, and Bd 3: ≥10 TBs; Figure 1a–c) [22]. Poorly differentiated clusters were also further classified based on the number of cells in each given cluster, thus yielding groups of PDCs made of 5–9 cells, 10–14 cells, and equal or more than 15 cells. PDC grades were determined similar to the TB grades based on the total number of PDCs (regardless of the number of constituting cells) in the investigated hot-spot area (PDC 0: 0, PDC 1: 1–4, PDC 2: 5–9, and PDC 3: ≥10 PDCs, respectively; Figure 1d,e).

SARIFA is an area located at the invasion front in which a tumor gland or a cluster of at least 5 tumor cells are in direct contact with adipocytes without separating stroma. SARIFA was assessed on the same slide as the TBs and PDCs, according to the method described by Grosser et al. [11]. The evaluation is based on the presence of SARIFA anywhere on the slide (SARIFA+) or the absence of this (SARIFA−; Figure 1f). 

### 2.3. Ethical Approval

The study protocol followed the ethical guidelines of the 1975 Declaration of Helsinki and was approved by the Ethical Committee of Semmelweis University, Budapest (SE-RKEB 242-1/2021). Based on the current Hungarian law for scientific research, contacting the patients in order to obtain their informed consent is not required for retrospective studies. According to this, the Ethical Committee of Semmelweis University, Budapest, waived the informed consent procedure for this study. The study complies with the REMARK checklist of reporting recommendations for tumor marker prognostic studies.

### 2.4. Statistical Analysis

All calculations and plotting were carried out in the R software environment (version 4.0.2). As in previous studies, the Bd 0, Bd 1, and Bd 2 groups were combined into the “TB low” category, while the Bd 3 group alone represented the “TB high” category for the calculations. Similarly, PDC counts were grouped into “PDC low” (PDC 0, 1, and 2) and “PDC high” (PDC 3) categories. Similar to other studies, for the calculation of hazard/odds ratios in multivariable analysis, a two-tier reclassification of the variable categories was used in order to reach the clinicopathologically most significant dichotomous separation of the cases (i.e., low/high grade), which provides greater statistical power and is a commonly used approach in clinical practice. Accordingly, instead of using all available increments in the calculations, pT stages I and II, as well as III and IV, were combined, resulting in a two-tiered scale (I–II vs. III–IV). Similarly, the pN stages were combined into a two-tier scheme (pN0 vs. pN+). For the statistical analysis, continuous variables were expressed by their mean, range, and standard deviation (SD). Categorical variables were described as frequencies, and their raw data were plotted in 2 × 2 contingency tables and analyzed using Fisher’s exact probability test. To find independent risk factors of lymph node metastases, univariable and multivariable logistic regression analysis with backward selection was applied. Correlation of the number and size of independent tumor cell clusters and SARIFA status was performed using the Mann–Whitney test. Survival analyses were performed using the Kaplan–Meier/log-rank method, and the correlation between overall survival and the absolute number of tumor buds, poorly differentiated clusters, and SARIFA+/− statuses were analyzed using age-adjusted Cox proportional hazards regression. All *p*-values were calculated as two-tailed and considered significant when *p* < 0.05.

## 3. Results

### 3.1. Cohort Characteristics and Differences in Clinicopathological Parameters of ESQCC and EAC Cases

A total of 100 patient cases were eligible for the study, including 80 males and 20 females. The mean age was 64.8 years (range 45–80). The histological subtype was squamous cell carcinoma in 57 cases and adenocarcinoma in 43 cases. Although lymphovascular invasion was observable in only 24 cases, lymph node metastasis was present in 47 cases. This matches the number of cases where perineural invasion could be observed. The mean lymph node ratio (number of metastatic lymph nodes divided by the number of all observed lymph nodes per case) was 0.15. The number of patients who received neoadjuvant therapy (either chemo-, radio-, or chemo-radiotherapy) was 70, out of which 43 patients were responders. Based on our results, patient age was significantly higher in EAC than in ESQCC (mean age: 66.7 vs. 63.4 years, *p* = 0.014). Furthermore, the presence of lymph node metastases (present vs. absent, 19 vs. 38 in ESQCC and 28 vs. 15 in EAC, *p* = 0.023), lymphovascular invasion (present vs. absent, 9 cases vs. 48 cases in ESQCC and 15 cases vs. 28 cases in EAC, *p* = 0.0342), perineural invasion (present vs. absent, 4 cases vs. 53 cases in ESQCC and 20 cases vs. 23 cases in EAC, *p* ≤ 0.0001), LNR (0.10 in ESQCC and 0.23 in EAC, *p* = 0.0080), and SARIFA (present vs. absent, 19 cases vs. 38 cases in ESQCC and 27 cases vs. 16 cases in EAC, *p* = 0.0046) was significantly higher in adenocarcinomas than in squamous cell carcinomas. However, regarding TB and PDC status, no significant difference was found between the two histological subtypes (Table 1).

### 3.2. Association between the Size of the Tumor Cell Clusters and SARIFA Status

**Figure 2:**
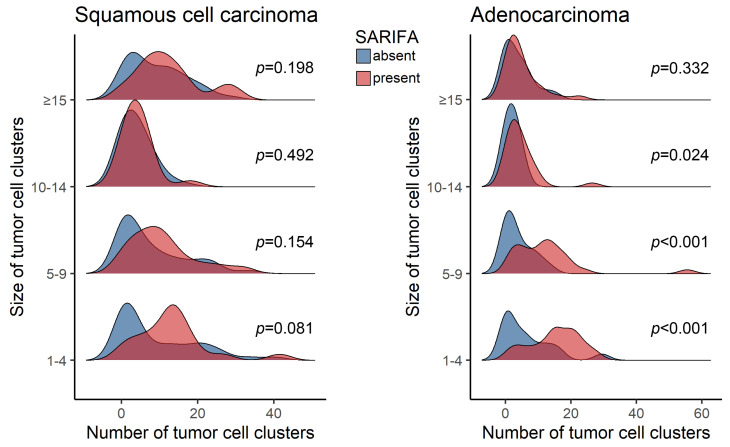
Number of tumor cell clusters of different sizes in SARIFA-positive and negative cases. Abbreviation: SARIFA—Stroma AReactive Invasion Front Area.

To characterize the relationship between tumor budding/PDC and SARIFA within the same tumor, the distribution of the number of tumor cell clusters (either TB or PDC, i.e., combining these two size categories into one spectrum) was plotted according to their size in SARIFA-positive and negative cases. In those adenocarcinomas where small clusters of cells occurred in large numbers (TBs and PDCs of few tumor cells), the presence of SARIFA was found to be significantly more frequent than in the subpopulation where small cell clusters occurred in small numbers (clusters of 1–4 tumor cell(s): *p* < 0.001, 5–9 tumor cells: *p* < 0.001, 10–14 tumor cells: *p* = 0.024, and ≥15 tumor cells: *p* = 0.332; Figure 2). This correlation was not observable in squamous cell carcinomas (Figure 2).

### 3.3. Association between TB/PDC/SARIFA Status and the Extension of the Tumor

In adenocarcinomas, tumor budding and the presence of SARIFA were correlated with greater tumor extension (*p* = 0.0162 and *p* = 0.0012, respectively). However, PDC status showed no significant correlation with the tumor extent. In the case of squamous cell carcinomas, no significant correlation was observed between TB, PDC, or SARIFA status and T stage (Table 2).

### 3.4. Association of TB/PDC/SARIFA Status with the Presence of Lymph Node Metastases

The presence of lymph node metastases was examined among patients with low and high TB, low and high PDC, and SARIFA-positive and negative status, respectively, for both squamous cell carcinomas and adenocarcinomas (Table 3). Univariable logistic regression showed a significant positive correlation between lymph-node-positive status and the low/high TB category (*p* = 0.0006) and the presence of vascular invasion (*p* = 0.0352) in squamous cell carcinomas. Multivariable analysis revealed that the lymph-node-positive status was significantly associated with a higher T stage (*p* = 0.0424) and the high TB status (*p* = 0.0006); thus, besides stage, tumor budding is also an independent prognostic factor for lymph node metastasis in squamous cell cancers. In adenocarcinomas, univariable regression showed that only SARIFA status was associated with lymph node positivity (*p* = 0.0054). Multivariable analysis also revealed a significant positive association (*p* = 0.0111), thus confirming that SARIFA positivity is an independent prognostic factor for lymph node metastasis in esophageal adenocarcinomas (Table 3).

### 3.5. Survival Analysis

**Figure 3:**
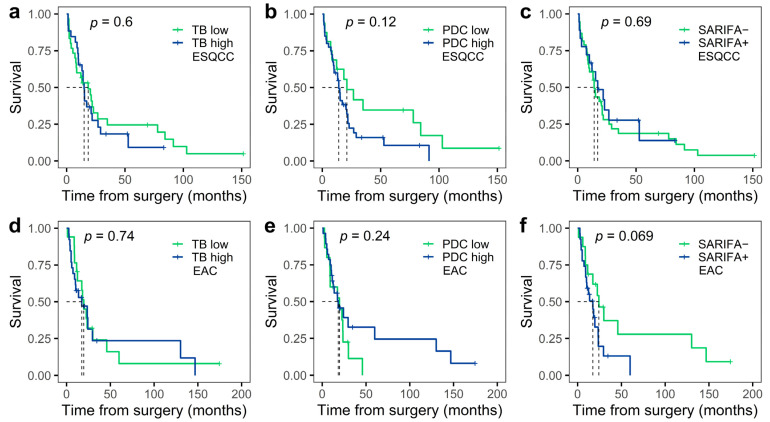
Kaplan–Meier curves showing overall survival for subgroups with TB low/high, PDC low/high, and SARIFA+/− status. TB low/high in squamous cell carcinoma (**a**), PDC low/high in squamous cell carcinoma (**b**), SARIFA+/− in squamous cell carcinoma (**c**), TB low/high in adenocarcinoma (**d**), PDC low/high in adenocarcinoma (**e**), and SARIFA+/− in adenocarcinoma (**f**). The dashed lines in the figure show median survival. Abbreviations: TB, tumor budding; PDC, poorly differentiated cluster; SARIFA, Stroma AReactive Invasion Front Area; ESQCC, esophageal squamous cell carcinoma; EAC, esophageal/esophagogastric junction adenocarcinoma.

The effect of TB low/high, PDC low/high, and SARIFA+/− status on overall survival was first analyzed by Kaplan–Meier curves and the log-rank test. No significant association was observed in squamous cell cancers, nor in adenocarcinomas (Figure 3).

To investigate this further, an age-adjusted Cox regression model for overall survival was applied using the absolute numbers of TBs and PDCs counted in each case as continuous variables. In squamous cell cancers, both TB (*p* = 0.0269) and PDC (*p* = 0.0377) showed a statistically significant negative association with overall survival, but no such relationship was found with SARIFA. In adenocarcinomas, neither of the aforementioned factors showed a significant association, but in the case of SARIFAs, a similar trend was observable, without reaching the threshold of significance (*p* = 0.0658; Table 4).

## 4. Discussion

In the age of personalized medicine, the presence of biomarkers that offer cost-effective, reliable, and quickly assessable prognostic factors for the determination of the disease course is a major field of interest in tumor pathology. In our study, we assessed the TB, PDC, and SARIFA status of 100 esophageal squamous cell cancers and adenocarcinomas, with a special emphasis on their influence regarding the development of lymphatic metastases.

According to the ITBCC protocol, the evaluation of TBs in colorectal cancer was introduced in the guidelines of the UICC TNM [5,19]. Several studies have examined their role in the upper gastrointestinal cancers, including gastric cancer, on which our group published a study on the role of TBs and PDCs [8,9]. Tumor budding resulting in the presence of TBs and PDCs on a classical two-dimensional histology slide is the visible manifestation of the epithelial–mesenchymal transition. However, the results of Bronsert et al.’s 3D microscopic reconstruction of tumor invasion showed that the process of tumor budding resembles a branching pattern, with tumor cells eventually detaching from the main tumor mass [23]. These results suggest that the two-dimensional units of TBs and PDCs are in fact cross-sections of these invasive tumor branches [23].

To our knowledge, this is the first study to directly compare the invasive pattern of esophageal squamous cell cancers and adenocarcinomas. Our results revealed a significantly higher rate of lymph node metastasis (*p* = 0.0023), lymphovascular (*p* = 0.0342) and perineural invasion (*p* < 0.0001), as well as SARIFA (*p* = 0.0046) in adenocarcinomas than in squamous cell carcinomas (Table 1). This is in contrast with the findings of Stein et al., where the lymphatic metastases were more frequent in early squamous cell esophageal cancers than in adenocarcinomas [24]. However, the underlying mechanism might offer an explanation regarding the difference, as the chronic inflammation in adenocarcinomas causes the superficial lymphatic vessels to occlude, but it does not affect the submucosal lymphatic vessels in the esophageal wall [24]. Our cohort included two times more advanced-stage adenocarcinomas compared to early-stage diseases, whereas in squamous cell carcinomas the number of advanced-stage diseases matched the number of early-stage tumors. This is also supported by the findings of Lagarde et al., since they found a significant positive correlation between higher pT stages and the higher frequency of lymphovascular invasion and lymph node metastases, and the number of adenocarcinomas was more than three times the number of squamous cell carcinomas in their cohort [25]. Accordingly, the proportion of early vs. more advanced adenocarcinomas in a given cohort may critically affect the occurrence of lymph node metastases, because the more advanced cancer is able to reach the deeper submucosal lymphatic vessels, which, unlike the more superficial ones, are not occluded by the chronic inflammatory infiltration that accompanies the tumor.

Since in the Western population the incidence of squamous cell esophageal carcinoma has showed a drastic decline [1], the proportion of squamous cell cancers in our cohort offer a unique opportunity to evaluate the significance of the aforementioned prognostic factors, in contrast to the continuously increasing number of esophageal adenocarcinomas [26].

Tumor budding/PDC [27] and/or SARIFA [13] have prognostic value regarding disease-free survival as well as overall survival in gastric cancer, which also suggests their potential utility in esophageal cancer. The usefulness in gastric cancer is supported by our findings of tumor budding being predictive of lymph node metastases [9], while Grosser et al. proved SARIFA to be predictive of overall survival [14,28].

Previous studies suggested that the prognostic value of SARIFA and TB depends on the extent of the tumor in gastrointestinal adenocarcinomas [11]. Although also found in pT1 cases, SARIFA more often occurs in already advanced cases, and our results support this theory. In line with this, in our cohort, advanced T stage was correlated with a high TB score (*p* = 0.01621) and SARIFA positivity (*p* = 0.001232) in patients with adenocarcinomas, while interestingly, a similar trend was not confirmed in squamous cell carcinoma cases (Table 2). Neoadjuvant therapy and its downstaging did not affect the distribution of TB/PDC/SARIFA status in this subgroup, compared with those not treated neoadjuvantly, and no significant difference was observed between those responding and not responding to neoadjuvant therapy either (see Supplementary Materials, Table S2).

The relationship between the two phenomena (tumor cell clusters of TB/PDC and SARIFA) has been less studied, although it is clear that more disseminating tumor nests may indicate a greater chance of tumor cells reaching adipocytes directly. This was indeed supported by the study of Ulase and colleagues, as in a large cohort that included not only gastric but also gastroesophageal carcinomas, they found a very weak positive correlation between SARIFA status and tumor cell budding [13]. This is in concordance with the findings of Martin et al., the same group that originally described SARIFA. They also found a weak positive correlation between SARIFA and tumor budding, as well as SARIFA and poorly differentiated clusters [14]. Our results support the findings of these studies in that there was a significant correlation between SARIFA status and the large number of tumor cell clusters consisting of a small number of cells. Based on this, it can be concluded that since both TB/PDC and SARIFA are somewhat different invasion phenomena with distinct biological bases, they contribute in different ways to the prediction of prognosis in the same patient, and thus both tumor budding and SARIFA may have a place in the prognostic/predictive diagnostics of these tumors in the future. However, further studies are needed to clarify their relationship and their exact role in the prediction of tumor dissemination, especially since this finding was only observed in adenocarcinomas and not in squamous cell carcinomas of the esophagus.

Differences in the predictive value of invasion phenomena were also observed in other aspects between adenocarcinomas and squamous cell carcinomas. Namely, in our cohort, SARIFA positivity was found to be an independent prognostic factor for lymph node metastases in adenocarcinomas (*p* = 0.0111), but not in squamous cell carcinomas. On the other hand, high tumor budding proved to be an independent predictor for lymphatic metastases in squamous cell carcinomas (*p* = 0.0006), but not in adenocarcinomas. Prediction of the probable lymph node metastases has a great importance in early diseases that are manageable with endoscopic modalities, namely, endoscopic mucosal resection (EMR) and endoscopic submucosal dissection (ESD) [29]. In advanced cases, esophageal resection—either transthoracic or transhiatal—with lymph node dissection is the preferred therapeutic approach [30]. The probability of lymph node metastases increases as the tumor progresses deeper into the submucosa, toward the lymphatic vessels [24]. Predicting high probability of lymph node metastases from biopsy material or EMR/ESD specimens in esophageal cancers can help in clinical therapeutic decision-making. Namely, tumor-budding- and/or SARIFA-based prediction of lymph node metastases may be useful for selecting patients requiring surgical lymph node removal, and further oncological management in these cases may be modified accordingly. On the other hand, by evaluating tumor budding, PDC, and/or SARIFA in biopsy specimens, it may be possible to select those esophageal cancer patients for whom endoscopic, minimally invasive EMR/ESD surgery may be considered as curative therapy, instead of resection surgery. Since the quality of biopsy samples often makes it impossible to determine the depth of invasion, the need for additional markers available for preoperative assessment to help determine the therapeutic approach is dire. Recent studies have confirmed the preoperative prognostic value of TB in esophageal cancer [31]. However, in light of the above, further studies are still needed to define the tumor budding grades and SARIFA diagnostic criteria with ideal prognostic value for esophageal cancer at a given tumor stage for routine prognostic use.

Probably due to the small cohort size, no significant difference was found between low and high TB, low and high PDC, and SARIFA-negative and positive subgroups in overall survival, as assessed by Kaplan–Meier curves and the log-rank test, considering either squamous cell carcinomas or adenocarcinomas (Figure 3). However, when the numbers of TBs and PDCs were used as continuous variables in an age-adjusted Cox regression model, both TB and PDC showed a statistically significant negative association with overall survival in squamous cell cancers (Table 4), meaning that an increased number of tumor cell clusters was associated with shorter survival. In contrast, when examining adenocarcinomas with the same model, TB and PDC showed no association with survival, and although the difference between SARIFA-negative and positive tumors was close to the significance threshold of the *p*-value here, the difference was not significant (Table 4).

As in retrospective, single-center studies in general, this study also has some limitations. In this context, the small size of the cohort is particularly noteworthy, as the histological subtypes of esophageal cancer differed strongly in several aspects of the prognostic value of histologically observable invasion patterns, as described in detail above. This is further complicated by the fact that we studied three different histological invasion features—tumor budding, poorly differentiated clusters, and SARIFA—two of which (TB and PDC) belong to the same phenotypic spectrum. Thus, less pronounced effects would probably only yield a statistically significant difference for larger numbers of cases, as would the effect of SARIFA on overall survival in adenocarcinomas. However, this also has the advantage that differences that prove statistically significant in such relatively small cohorts are also likely to have strong biological significance. Nevertheless, independent prospective studies with a substantially larger number of patients are expected to confirm our results beyond a reasonable doubt. A further issue was that following the ITBCC protocol for tumor budding and PDC evaluation, no additional immunohistochemical staining was used to visualize tumor cell clusters. However, under certain circumstances, the accurate histological identification of tumor cell nests may be facilitated by immunohistochemical labeling of tumor cells [5]. Previous studies have shown that the immunohistochemical marker maspin is able to label not only tumor budding cells but also those “at the point of budding”. The results of our study may be limited by the fact that we did not use such a marker, which could have been used both to clarify the SARIFA–budding relationship and to more accurately estimate the risk of lymph node metastasis [32].

## 5. Conclusions

As a summary, our results suggested that the histological subtypes of esophageal cancer behave differently, namely, that different features of the invasion front are of prognostic significance. We found that SARIFA positivity was significantly more frequent in esophageal adenocarcinomas than in squamous cell carcinomas, and the prognostic value of SARIFA positivity in esophageal adenocarcinomas was highlighted by a positive correlation with the presence of lymph node metastases, which was proven to be an independent prognostic factor in the multivariable model. To our knowledge, our group was the first to test this promising biomarker on the squamous cell carcinomas of the esophagus. Unfortunately, our results did not establish SARIFA as a useful prognostic marker in esophageal squamous cell cancers.

On the other hand, in one of our previous publications, we found tumor budding as a useful prognostic factor regarding overall survival and lymph node metastasis development in gastric adenocarcinomas [9]. In this current study, tumor budding was also found to be an independent prognostic factor for lymph node metastasis in esophageal squamous cell carcinomas. The fact that tumor budding in squamous cell cancers and SARIFA status in adenocarcinomas have been shown to be useful prognostic markers for predicting the development of lymph node metastases in esophageal cancer, and that in squamous cell carcinoma the higher absolute number of both TBs and PDCs has been associated with shorter overall survival, further broadens the spectrum of oncological indications in which TB, PDC, and SARIFA can provide prognostic/predictive information.

## Figures and Tables

**Table 1:** Cohort characteristics and differences in clinicopathological parameters of ESQCC and EAC cases. Statistically significant results are highlighted in bold. Abbreviations: pT, pathological tumor (T) stage; pN, pathological lymph node (N) stage; pM, pathological distant metastasis (M) stage; LNR, lymph node ratio; PDC, poorly differentiated cluster; SARIFA, Stroma AReactive Invasion Front Area. * Grade was determined only for cases without previous neoadjuvant therapy. ** Mandard score was determined only for cases with previous neoadjuvant therapy.

Cohort Characteristics		Total	Squamous Cell Carcinoma	Adenocarcinoma	*p*
Patients	*n*	100	57	43	-
Age	*year*	64.8	63.4 (45–80)	66.7 (42–80)	**0.0140**
Sex	*male*	80	42 (73.7%)	38 (88.4%)	0.0815
	*female*	20	15 (26.3%)	5 (11.6%)	
pT	*low*	41	28 (49.1%)	13 (30.2%)	0.0670
	*high*	59	29 (50.9%)	30 (69.8%)	
pN	*absent*	53	38 (66.7%)	15 (34.9%)	**0.0023**
	*present*	47	19 (33.3%)	28 (65.1%)	
pM	*absent*	97	56 (98.2%)	42 (97.7%)	1
	*present*	3	1 (1.8%)	1 (2.3%)	
Vascular invasion	*absent*	78	44 (77.2%)	34 (79.1%)	1
	*present*	22	13 (22.8%)	9 (20.9%)	
Lymphovascular invasion	*absent*	76	48 (84.2%)	28 (65.1%)	**0.0342**
	*present*	24	9 (15.8%)	15 (34.9%)	
Perineural invasion	*absent*	76	53 (93.0%)	23 (53.5%)	**<0.0001**
	*present*	24	4 (7.0%)	20 (46.5%)	
Resection margin	*tumor-free*	78	46 (80.7%)	32 (74.4%)	0.4746
	*positive*	22	11 (19.3%)	11 (25.6%)	
LNR	*mean*	0.15	0.10	0.23	**0.0080**
Grade *	*low*	16	11 (64.7%)	5 (38.5%)	1
	*high*	14	6 (35.3%)	8 (61.5%)	
Necrosis	*absent*	57	29 (50.9%)	28 (65.1%)	0.2208
	*present*	43	28 (49.1%)	15 (34.9%)	
Neoadjuvant therapy	*no*	30	17 (29.8%)	13 (30.2%)	1
	*yes*	70	40 (70.2%)	30 (69.8%)	
Mandard score **	*responder*	43	26 (65.0%)	17 (56.7%)	0.6204
	*non-responder*	27	14 (35.0%)	13 (43.3%)	
Tumor budding	*low*	47	30 (52.6%)	17 (39.5%)	0.2278
	*high*	53	27 (47.4%)	26 (60.5%)	
PDC	*low*	31	16 (28.1%)	15 (34.9%)	0.5165
	*high*	69	41 (71.9%)	28 (65.1%)	
SARIFA	*absent*	54	38 (66.7%)	16 (37.2%)	**0.0046**
	*present*	46	19 (33.3%)	27 (62.8%)	

**Table 2:** Association between TB/PDC/SARIFA status and the tumor T stage. Statistically significant results are highlighted in bold. Abbreviations: TB, tumor budding; PDC, poorly differentiated cluster; SARIFA, Stroma AReactive Invasion Front Area.

Squamous Cell Carcinoma		Total	T-Low	T-High	*p*
TB	low	26	12	14	0.7921
	high	31	16	15	
PDC	low	16	11	5	0.0819
	high	41	17	24	
SARIFA	absent	38	21	17	0.2630
	present	19	7	12	
**Adenocarcinoma**		**Total**	**T-Low**	**T-High**	***p***
TB	low	17	9	8	**0.0162**
	high	26	4	22	
PDC	low	15	6	9	0.3238
	high	28	7	21	
SARIFA	absent	16	10	6	**0.0012**
	present	27	3	24	

**Table 3:** Univariable and multivariable analyses for unveiling the association between TB/PDC/SARIFA status and the presence of lymph node metastases in esophageal squamous cell carcinomas (A) and adenocarcinomas (B). Statistically significant results are highlighted in bold. Abbreviations: TB, tumor budding; PDC, poorly differentiated cluster; SARIFA, Stroma AReactive Invasion Front Area; n.s., not significant.

(A)					
Parameter		Squamous Cell Carcinoma			
		Univariable		Multivariable	
		OR (CI)	*p*	OR (CI)	*p*
Age	*years*	1.007 (0.935–1.087)	0.8430		n.s.
Sex	*male/female*	0.491 (0.0995–1.872)	0.3270		n.s.
TB	*low/high*	12.273 (3.291–61.133)	**0.0006**	25.32 (4.88–222.0)	**0.0006**
PDC	*low/high*	4.667 (1.103–32.334)	0.0609		n.s.
T stage	*low/high*	2.750 (0.876–9.390)	0.0909	6.035 (1.244–46.336)	**0.0424**
SARIFA	*absent/present*	1.083 (0.314–3.534)	0.8956		n.s.
Vascular invasion	*absent/present*	4.20 (1.123–16.941)	**0.0352**		n.s.
Lymphovascular invasion	*absent/present*	1.886 (0.414–8.198)	0.3931		n.s.
Perineural invasion	*absent/present*	7.40 (0.870–156.349)	0.0938		n.s.
Resection margin	*tumor-free/* *positive*	0.886 (0.172–3.696)	0.8730		n.s.
Neoadjuvant therapy	*no/yes*	2.60 (0.697–12.694)	0.1839		n.s.
**(B)**					
**Parameter**		**Adenocarcinoma**			
		**Univariable**		**Multivariable**	
		**OR (CI)**	***p***	**OR (CI)**	***p***
Age	*years*	1.025 (0.961–1.094)	0.4460		n.s.
Sex	*male/female*	0.780 (0.115–6.486)	0.7988		n.s.
TB	*low/high*	2.413 (0.671–9.054)	0.1800		n.s.
PDC	*low/high*	2.188 (0.592–8.276)	0.2390		n.s.
T stage	*low/high*	3.208 (0.834–13.047)	0.0924		n.s.
SARIFA	*absent/present*	7.333 (1.903–32.532)	**0.0054**	11.0 (2.0–91.78)	**0.0111**
Vascular invasion	*absent/present*	5.600 (0.881–109.986)	0.1230		n.s.
Lymphovascular invasion	*absent/present*	362,730,000 (<0.0001-Infinity)	0.9940		n.s.
Perineural invasion	*absent/present*	3.667 (0.987–15.955)	0.0626		n.s.
Resection margin	*tumor-free/* *positive*	3.079 (0.656–22.413)	0.1910		n.s.
Neoadjuvant therapy	*no/yes*	0.768 (0.175–2.989)	0.7100		n.s.

**Table 4:** Analysis of the association of overall survival with the absolute numbers of TBs and PDCs, as well as SARIFA status in an age-adjusted Cox proportional hazards regression model. Statistically significant results are highlighted in bold. Abbreviations: ESQCC, esophageal squamous cell carcinoma; PDC, poorly differentiated cluster; SARIFA, Stroma AReactive Invasion Front Area; EAC, esophageal adenocarcinoma.

ESQCC	OR	CI	*p*
Tumor budding	1.036007	1.0040570–1.068972	**0.0269**
Age	0.994523	0.9533010–1.037527	0.7993
PDC	1.018223	1.0010303–1.035712	**0.0377**
Age	0.998127	0.9571506–1.040857	0.9301
SARIFA	0.872240	0.4520001–1.683178	0.6840
Age	1.001310	0.9599019–1.044505	0.9520
**EAC**	**OR**	**CI**	***p***
Tumor budding	1.008108	0.9628542–1.055489	0.7300
Age	1.029528	0.9921378–1.068327	0.1230
PDC	0.978292	0.9467089–1.010929	0.1900
Age	1.026867	0.9914605–1.063539	0.1390
SARIFA	2.098440	0.9527029–4.622074	0.0658
Age	1.032400	0.9929269–1.073441	0.1089

## Data Availability

The authors declare that the data supporting the findings of this study are available within the paper and its Supplementary Materials. Further inquiries can be directed to the corresponding authors.

## Supplementary Materials

The following supporting information can be downloaded at: https://www.mdpi.com/article/10.3390/cancers16183144/s1. Table S1: Additional clinical and pathological data. Table S2: Impact of TB low/high, PDC low/high, and SARIFA+/− status on neoadjuvant therapy outcomes in EAC (A) and ESQCC (B).
