# Supplementary material for: Local Invasion Patterns Characterized by SARIFA and Tumor Budding Differ and Have Distinct Prognostic Significance in Esophageal Adenocarcinoma and Squamous Cell Carcinoma
Source: Cancers (Basel). 2024 Sep 13;16(18):3144. doi: 10.3390/cancers16183144 (PMC11430286; doi:10.3390/cancers16183144)
Supplement: Supplementary file 1 [file cancers-16-03144-s001.zip › cancers-3177200-supplementary.pdf]

cancers MDPI

Supplementary Materials

Local Invasion Patterns Characterized by SARIFA and Tumor Budding Differ and Have Distinct Prognostic Significance in Esophageal Adenocarcinoma and Squamous Cell Carcinoma

Ákos Jakab [1], Levente Zarándy [1], Ildikó Kocsmár [2], Tibor Várkonyi [1], István Kenessey [1,3], Attila Szijártó [4], András Kiss [1], Tamás Vass [4], Gábor Lotz [1,*,†] and Éva Kocsmár [1,*,†]

[1] Department of Pathology, Forensic and Insurance Medicine, Semmelweis University, Üllői Str. 93, H-1091 Budapest, Hungary; jakab.akos@stud.semmelweis.hu (Á.J.); zarandy.levente@stud.semmelweis.hu (L.Z.); varkonyi.tibor1@semmelweis.hu (T.V.); kenessey.istvan@oncol.hu (I.K.); kiss.andras@semmelweis.hu (A.K.)
[2] Department of Urology, Semmelweis University, Üllői Str. 78/b, H-1082 Budapest, Hungary; kocsmar.ildiko@semmelweis.hu
[3] National Cancer Registry, National Institute of Oncology, Ráth György Str. 7-9, H-1122 Budapest, Hungary
[4] Department of Surgery, Transplantation and Gastroenterology, Semmelweis University, Üllői Str. 78, H-1082 Budapest, Hungary; szijarto.attila@semmelweis.hu (A.S.); vass.tamas@semmelweis.hu (T.V.)
* Correspondence: lotz.gabor@med.semmelweis-univ.hu (G.L.); kocsmar.eva@semmelweis.hu (É.K.)
† These authors contributed equally to this work.

Table S1. Additional clinical and pathological data. CHRTH: chemoradiotherapy, * including patients received neoadjuvant therapy, ** including patients received adjuvant therapy.

Variable		n / %
Number of patients	n	100
Age at surgery (years)	mean, range	64.82 (42-80)
Sex	male	80 (80%)
	female	20 (20%)
pT (/ ypT)	1a	7 (7%)
	1b	8 (8%)
	2	26 (26%)
	3	55 (55%)
	4a	4 (4%)
	4b	0 (0%)
pN (/ ypN)	0	53 (53%)
	1	28 (28%)
	2	7 (7%)
	3	12 (12%)
pM	0	97 (97%)
	1	3 (3%)
Type of esophagectomy	transthoracal	33 (33%)
	transhiatal	57 (57%)
	distal esophagectomy with gastrectomy	8 (8%)
	other	2 (2%)
Neoadjuvant therapy	yes	70 (70%)
	no	30 (30%)
Neoadjuvant therapy regimen*	CROSS	9 (9%)
	FLOT	12 (12%)
	CHRTH, not specified	39 (39%)

	chemotherapy - individual protocol	10 (10%)
Mandard score	2	17 (17%)
	3	14 (14%)
	4	17 (17%)
	5	22 (22%)
Adjuvant therapy	yes	38 (38%)
	no	40 (40%)
	not known	22 (22%)
Adjuvant therapy regimen**	FLOT	6 (6%)
	CHRTH - individual protocol	4 (4%)
	chemotherapy - individual protocol	6 (6%)
	received, not specified	22 (22%)
Follow-up period	mean, range	24.99 (1-174)
Deaths	n	79 (79%)

Table S2. Impact of TB low/high, PDC low/high and SARIFA +/- status on neoadjuvant therapy outcome in EAC (A) and ESQCC (B). Abbreviations: ESQCC: esophageal squamous cell carcinoma, TB: tumor budding, PDC: poorly differentiated cluster, SARIFA: stroma areactive invasion front area, EAC: esophageal adenocarcinoma, Neo: patient received neoadjuvant chemo- or chemo-radiotherapy, Nonneo: patient received no neoadjuvant therapy, Resp: therapy responder, Nonresp: non-responder.

**A)**	**EAC**	**Total**	**Neo**	**Nonneo**	***p***
TB	low	17	10	7	0.3098
	high	26	20	6	
PDC	low	15	11	4	1
	high	28	19	9	
SARIFA	absent	16	11	5	1
	present	27	19	8	
	**EAC with Neo**	**Total**	**Resp**	**Nonresp**	***p***
TB	low	10	8	2	0.4195
	high	20	12	8	
PDC	low	11	9	2	0.2465
	high	19	11	8	
SARIFA	absent	11	9	2	0.2465
	present	19	11	8	

**B)**	**ESQCC**	**Total**	**Neo**	**Nonneo**	***p***
TB	low	30	20	10	0.5761
	high	27	20	7	
PDC	low	16	11	5	1
	high	41	29	12	
SARIFA	absent	38	26	12	0.7662
	present	19	14	5	
	**ESQCC with Neo**	**Total**	**Resp**	**Nonresp**	***p***
TB	low	20	15	5	1
	high	20	14	6	
PDC	low	11	10	1	0.2326
	high	29	19	10	

SARIFA	absent	26	19	7	1
	present	14	10	4	
